# Supplementary material for: Estimating the potato farming efficiency: A comparative study between stochastic frontier analysis and data envelopment analysis
Source: PLoS One. 2023 Apr 13;18(4):e0284391. doi: 10.1371/journal.pone.0284391 (PMC10101415; doi:10.1371/journal.pone.0284391)
Supplement: S1 Table — (DOCX) [file pone.0284391.s001.docx]

**S1 Table:** Frequency distribution (%) of farms according to technical and scale efficiencies

| **Efficiency Index (%)** | **Technical Efficiency (TE)** | | **Scale Efficiency (SE)** |
| --- | --- | --- | --- |
|  | **CRS (Overall TE)** | **VRS (Pure TE)** |  |
| 01-40 | 52 | 35 | 4 |
| 40-50 | 17 | 21 | 3 |
| 50-60 | 12 | 17 | 4 |
| 60-70 | 6 | 6 | 6 |
| 70-80 | 4 | 5 | 9 |
| 80-90 | 3 | 3 | 20 |
| 90-100 | 6 | 13 | 54 |
| Total farms | 300 | | |
| **Summary Statistics** | | | |
| Mean | 43.76 | 52.71 | 84.50 |
| Minimum | 7.4 | 15.9 | 7.4 |
| Maximum | 100 | 100 | 100 |
| Standard Deviation | 21.49 | 23.12 | 18.32 |
