# Supplementary material for: Estimating the potato farming efficiency: A comparative study between stochastic frontier analysis and data envelopment analysis
Source: PLoS One. 2023 Apr 13;18(4):e0284391. doi: 10.1371/journal.pone.0284391 (PMC10101415; doi:10.1371/journal.pone.0284391)
Supplement: S2 Table — (DOCX) [file pone.0284391.s002.docx]

**S2 Table:** Frequency distribution (%) of efficiency for farms

| **Efficiency Index (%)** | **SFA** | | | **DEA** | | | | | |
| --- | --- | --- | --- | --- | --- | --- | --- | --- | --- |
|  |  |  |  | **CRS** | | | **VRS** | | |
|  | TE | AE | EE | TE | AE | EE | TE | AE | EE |
| 01-40 | 0 | 1 | 3 | 24 | 8 | 43 | 20 | 11 | 44 |
| 40-50 | 2 | 4 | 16 | 8 | 8 | 2 | 6 | 7 | 4 |
| 50-60 | 3 | 10 | 18 | 5 | 17 | 5 | 7 | 15 | 5 |
| 60-70 | 6 | 17 | 27 | 3 | 19 | 23 | 5 | 16 | 14 |
| 70-80 | 12 | 28 | 28 | 6 | 32 | 17 | 6 | 26 | 14 |
| 80-90 | 35 | 24 | 7 | 12 | 14 | 7 | 8 | 13 | 10 |
| 90-100 | 42 | 16 | 1 | 42 | 2 | 3 | 48 | 12 | 9 |
| Total Farms | 300 | | | | | | | | |
| **Summary Statistics** | | | | | | | | | |
| Mean | 86 | 75 | 64 | 71.04 | 65.56 | 48.08 | 74.52 | 67.92 | 51.77 |
| Minimum | 42 | 38 | 36 | 14.9 | 4.3 | 3.3 | 15.9 | 4.1 | 3.5 |
| Maximum | 100 | 100 | 95 | 100 | 98.5 | 98.5 | 100 | 100 | 100 |
| Standard Deviation | 12 | 14 | 12 | 29.73 | 16.91 | 26.50 | 28.76 | 18.89 | 28.02 |
