# Supplementary material for: Estimating the potato farming efficiency: A comparative study between stochastic frontier analysis and data envelopment analysis
Source: PLoS One. 2023 Apr 13;18(4):e0284391. doi: 10.1371/journal.pone.0284391 (PMC10101415; doi:10.1371/journal.pone.0284391)
Supplement: Attachment — Submitted filename: PONE-D-23-04113 (1).pdf [file pone.0284391.s004.pdf]

PLOS ONE

Estimating the Potato Farming Efficiency: A Comparative Study between Stochastic Frontier Analysis and Data Envelopment Analysis

--Manuscript Draft--

**Manuscript Number:**	PONE-D-23-04113
**Article Type:**	Research Article
**Full Title:**	Estimating the Potato Farming Efficiency: A Comparative Study between Stochastic Frontier Analysis and Data Envelopment Analysis
**Short Title:**	Estimating the Potato Farming Efficiency
**Corresponding Author:**	Md. Moyazzem Hossain<br>Jahangirnagar University<br>Savar, Dhaka, BANGLADESH
**Keywords:**	Potato Farming; Efficiency; Stochastic Frontier Analysis; Data Envelopment Analysis
**Abstract:**	The government of Bangladesh has been trying to encourage potato consumption to reduce pressure on rice consumption and earn foreign currency along with ensuring zero hunger that helps to achieve the Sustainable Development Goal. It is necessary to use farmers' resources and current technology more efficiently to meet the demand. This study evaluates farm-level efficiency and inefficiency of potato farming by estimating technical, allocative, and economic efficiencies using the Cobb-Douglas Stochastic Frontier Analysis (SFA) and the input-oriented Data Envelopment Analysis (DEA) method. The primary data were collected through interviews of 300 potato farmers from Munshigonj, Rangpur, Dinajpur, and Joypurhat districts of Bangladesh. The findings revealed that the efficiency score of the SFA model is higher than the DEA model, which implies that the SFA frontier fits better than the DEA frontier. In the case of DEA, variable returns to scale (VRS) technical efficiency (TE) enveloped data more closely than constant returns to scale (CRS) TE. Results of efficiency suggest significant economic, technical, and allocative inefficiencies in potato farming and there is a scope to increase potato production levels through efficiency improvement. Inefficiency analysis shows that infrastructure and socio-economic factors jointly influence potato production variability. The authors suggest for using the Stochastic Frontier Analysis to find efficiencies in the agriculture sector. To achieve efficiency in potato production, the government needs to pay attention for improving the allocative and economic efficiencies along with emphasizing to choose the appropriate technology and efficient use of resources for the scale of operation.
**Order of Authors:**	Shamima Sultana, PhD
	Md. Moyazzem Hossain, MPhil
	Md. Nurul Haque, PhD
**Additional Information:**	
**Question**	**Response**
**Financial Disclosure**<br><br>Enter a financial disclosure statement that describes the sources of funding for the work included in this submission. Review the submission guidelines for detailed requirements. View published research articles from *PLOS ONE* for specific examples.<br><br>This statement is required for submission and **will appear in the published article** if	The author(s) received no specific funding for this work.

Powered by Editorial Manager® and ProduXion Manager® from Aries Systems Corporation

the submission is accepted. Please make sure it is accurate.<br><br>**Unfunded studies**<br>Enter: *The author(s) received no specific funding for this work.*<br><br>**Funded studies**<br>Enter a statement with the following details:<br>• Initials of the authors who received each award<br>• Grant numbers awarded to each author<br>• The full name of each funder<br>• URL of each funder website<br>• Did the sponsors or funders play any role in the study design, data collection and analysis, decision to publish, or preparation of the manuscript?<br>• **NO** - Include this sentence at the end of your statement: *The funders had no role in study design, data collection and analysis, decision to publish, or preparation of the manuscript.*<br>• **YES** - Specify the role(s) played.<br><br>* typeset	
**Competing Interests**<br><br>Use the instructions below to enter a competing interest statement for this submission. On behalf of all authors, disclose any competing interests that could be perceived to bias this work—acknowledging all financial support and any other relevant financial or non-financial competing interests.<br><br>This statement is required for submission and **will appear in the published article** if the submission is accepted. Please make sure it is accurate and that any funding sources listed in your Funding Information later in the submission form are also declared in your Financial Disclosure statement.<br><br>View published research articles from *PLOS ONE* for specific examples.	The authors have declared that no competing interests exist.

**NO authors have competing interests**<br><br>Enter: *The authors have declared that no competing interests exist.*<br><br>**Authors with competing interests**<br><br>Enter competing interest details beginning with this statement:<br><br>*I have read the journal's policy and the authors of this manuscript have the following competing interests: [insert competing interests here]*<br><br>* typeset	
**Ethics Statement**<br><br>Enter an ethics statement for this submission. This statement is required if the study involved:<br><br>• Human participants<br>• Human specimens or tissue<br>• Vertebrate animals or cephalopods<br>• Vertebrate embryos or tissues<br>• Field research<br><br>Write "N/A" if the submission does not require an ethics statement.<br><br>General guidance is provided below. Consult the submission guidelines for detailed instructions. **Make sure that all information entered here is included in the Methods section of the manuscript.**	Before participating in the survey, the authors explained the purpose of the study to the participants and assured them that their answers would be kept private, that no personally identifiable information would be shared, and that their verbal consent would be obtained.

Powered by Editorial Manager® and ProduXion Manager® from Aries Systems Corporation

**Format for specific study types**<br><br>**Human Subject Research (involving human participants and/or tissue)**<br>• Give the name of the institutional review board or ethics committee that approved the study<br>• Include the approval number and/or a statement indicating approval of this research<br>• Indicate the form of consent obtained (written/oral) or the reason that consent was not obtained (e.g. the data were analyzed anonymously)<br><br>**Animal Research (involving vertebrate animals, embryos or tissues)**<br>• Provide the name of the Institutional Animal Care and Use Committee (IACUC) or other relevant ethics board that reviewed the study protocol, and indicate whether they approved this research or granted a formal waiver of ethical approval<br>• Include an approval number if one was obtained<br>• If the study involved *non-human primates*, add *additional details* about animal welfare and steps taken to ameliorate suffering<br>• If anesthesia, euthanasia, or any kind of animal sacrifice is part of the study, include briefly which substances and/or methods were applied<br><br>**Field Research**<br><br>Include the following details if this study involves the collection of plant, animal, or other materials from a natural setting:<br>• Field permit number<br>• Name of the institution or relevant body that granted permission	
**Data Availability**<br><br>Authors are required to make all data underlying the findings described fully available, without restriction, and from the time of publication. PLOS allows rare exceptions to address legal and ethical concerns. See the PLOS Data Policy and FAQ for detailed information.	No - some restrictions will apply

A Data Availability Statement describing where the data can be found is required at submission. Your answers to this question constitute the Data Availability Statement and **will be published in the article**, if accepted.<br><br>**Important:** Stating 'data available on request from the author' is not sufficient. If your data are only available upon request, select 'No' for the first question and explain your exceptional situation in the text box.<br><br>Do the authors confirm that all data underlying the findings described in their manuscript are fully available without restriction?	
**Describe where the data may be found in full sentences. If you are copying our sample text, replace any instances of XXX with the appropriate details.**<br><br>• If the data are **held or will be held in a public repository**, include URLs, accession numbers or DOIs. If this information will only be available after acceptance, indicate this by ticking the box below. For example: *All XXX files are available from the XXX database (accession number(s) XXX, XXX.).*<br>• If the data are all contained **within the manuscript and/or Supporting Information files**, enter the following: *All relevant data are within the manuscript and its Supporting Information files.*<br>• If neither of these applies but you are able to provide **details of access elsewhere**, with or without limitations, please do so. For example:<br><br>*Data cannot be shared publicly because of [XXX]. Data are available from the XXX Institutional Data Access / Ethics Committee (contact via XXX) for researchers who meet the criteria for access to confidential data.*<br><br>*The data underlying the results presented in the study are available from (include the name of the third party*	All relevant data are within the manuscript and its Supporting Information files.

and contact information or URL).<br>• This text is appropriate if the data are owned by a third party and authors do not have permission to share the data.<br><br>* typeset	
Additional data availability information:	

Powered by Editorial Manager® and ProduXion Manager® from Aries Systems Corporation

Manuscript

Click here to access/download;Manuscript;Manuscript_SFA-DEA_12.02.2023.docx

Estimating the Potato Farming Efficiency: A Comparative Study between Stochastic Frontier Analysis and Data Envelopment Analysis

Shamima Sultana[1,*], Md. Moyazzem Hossain[2,*] and Md. Nurul Haque[3]

[1]Department of Economics, Jagannath University, Dhaka-1100, Bangladesh. Email: shamima@eco.jnu.ac.bd

[2]Department of Statistics, Jahangirnagar University, Dhaka-1342, Bangladesh, Email: hossainmm@juniv.edu

[3]Professor of Economics, Jahangirnagar University, Dhaka-1342, Bangladesh, Email: nhoque.econ@juniv.edu

Abstract

The government of Bangladesh has been trying to encourage potato consumption to reduce pressure on rice consumption and earn foreign currency along with ensuring zero hunger that helps to achieve the Sustainable Development Goal. It is necessary to use farmers' resources and current technology more efficiently to meet the demand. This study evaluates farm-level efficiency and inefficiency of potato farming by estimating technical, allocative, and economic efficiencies using the Cobb-Douglas Stochastic Frontier Analysis (SFA) and the input-oriented Data Envelopment Analysis (DEA) method. The primary data were collected through interviews of 300 potato farmers from Munshigonj, Rangpur, Dinajpur, and Joypurhat districts of Bangladesh. The findings revealed that the efficiency score of the SFA model is higher than the DEA model, which implies that the SFA frontier fits better than the DEA frontier. In the case of DEA, variable returns to scale (VRS) technical efficiency (TE) enveloped data more closely than constant returns to scale (CRS) TE. Results of efficiency suggest significant economic, technical, and allocative inefficiencies in potato farming and there is a scope to increase potato production levels through efficiency improvement. Inefficiency analysis shows that infrastructure and socio-economic factors jointly influence potato production variability. The authors suggest for using the Stochastic Frontier Analysis to find efficiencies in the agriculture sector. To achieve efficiency in potato production, the government needs to pay attention for improving the allocative and economic efficiencies

along with emphasizing to choose the appropriate technology and efficient use of resources for the scale of operation.

Keywords: Potato Farming; Efficiency; Stochastic Frontier Analysis; Data Envelopment Analysis

Introduction

The potato (Solanum tuberosum L.) is the most productive crop and the third-largest source of food on earth [1]. Despite being known as a rice-eating country, Bangladesh produces and consumes a lot of potatoes each year and it has steadily grown in popularity. Both rich and poor people use potatoes as a food crop and a vegetable. People eat cooked potatoes, fries, and chips [2]. After rice and wheat, potatoes are one of Bangladesh's key food crops. Over the past five years, the nation has produced potatoes at a rate of 10 million MT on average. Bangladesh is the fourth-largest producer of potatoes in Asia and is in the top 15 in the world [3]. The geographical suitability of Bangladesh is also responsible for the increased production of potatoes in every year [4]. However, they are vulnerable to climate change since the production of potatoes is extremely sensitive to a variety of abiotic factors, such as temperature and soil salinity [1,5].

Nowadays, developing countries' agricultural farming efficiency has been a topic of considerable interest in development literature. Efficiency is a performance measure and success indicator. The efficiency studies show the possibility of productivity raising through efficiency improvement without introducing new technologies or increasing the resource base. Farming efficiency of the agriculture sector is considered an essential factor in balancing population growth and agricultural output (food and raw materials). Inefficiency estimations help in taking a decision to raise productivity, whether through efficiency improvement or the introduction of new technology. In Bangladesh, potatoes have become a cash crop, and their importance is rising rapidly in the domestic and international markets. A previous study highlighted that ineffective transportation, inadequate storage, a shortage of capital, farmers' ignorance of market prices, illiteracy, and the syndicate system of middlemen are some of the major contributing causes to Bangladesh's ineffective potato marketing system [6]. Farmers' farming efficiency is an important tool for

optimizing production decisions and strengthening the farms' capacity to face increasing input costs, changing market conditions, rapid technological progress, and economic hardships to reach optimum output levels. Farmers' farming efficiency depends on their level of education, years of experience, land fragmentation, use of seeds, modern technology, fertilizer, and other inputs [7,8]. It is necessary to undertake research efforts to estimate the efficiency of potato farming to ensure better ways and sustainably improve potato productivity. Farmers need to improve their farming efficiency to achieve financial success and profit. There are variations in the resources used effectively in different farms. Resources should be utilized optimally to reach the maximum level of output and income. Farms' decision about raising farming efficiency depends on identifying inefficiency factors. Therefore, policymakers may give more emphasis on the improvement of efficiency. It is important to assess the efficiency of potato farming to find out the potential and possibilities for the expansion and sustainability of potato production in Bangladesh.

There is few information about the economic, technical, and allocative efficiencies of the potato sector in Bangladesh. Most studies estimate technical efficiency using SFA, however, some studies estimate economic, technical, and allocative efficiencies in the agriculture sector of Bangladesh using DEA and SFA [9–12]. There are several studies on the comparison between DEA and SFA for different crops in different countries [7,13–19]. To the best of our knowledge, no research has been done yet on potato farming efficiency using both SFA and DEA. The study's objectives are to estimate the potato farming efficiency (economic, technical, and allocative efficiencies), identify the explanatory factors of potato farming efficiency, and find any significant difference between the efficiency results of SFA and DEA methods. This study emphasizes for utilizing the allocated resources efficiently in potato farming which may help to improve the prevailing situation through developing policy parameters.

Methodology

Data and Variables

This study collected data from 300 individual farmers using a semi-structured questionnaire from four districts Munshigonj, Rangpur, Dinajpur, and Joypurhat of Bangladesh which is commercial potato-grown areas. Before participating in the survey, the authors explained the purpose of the study to the participants and assured them that their answers would be kept private, that no

personally identifiable information would be shared, and that their verbal consent would be obtained. This study collected the following variables from the survey: land (decimal), yield (kg), Seed (kg), tilling (Tk.), cost of labor (Tk.), irrigation (Tk.), fertilizer (Tk.), vitamin (Tk.), and pesticides (Tk.), in thousand where Tk. Is the currency of Bangladesh. Inefficiency factors such as education (year), age (year), experience (year), training (dummy), access to credit (dummy), land fragmentation (average plot size), weed uprooting cost (Tk.), household size (number of family members), and cold storage facility (dummy) are used in this study.

Methods of Estimation

Farrell's (1957) pioneering article on efficiency measurement helps develop different efficiency analysis methods. SFA and DEA are mostly used in efficiency-related research work worldwide [20]. Generally, SFA is used to assess the performance of the agricultural sector. DEA is used to estimate the efficiency of the non-agricultural sector, e.g., public utilities, banks, hospitals, education institutions, etc. However, the number of research carried out using DEA and SFA methods for crops. This research employs the SFA and DEA methods to estimate two districts' efficient and inefficient determinants. Two methods are discussed as follows:

Stochastic Frontier Analysis (SFA)

Aigner, et al. (1977) and Meeusen and van den Broeck (1977) independently suggested the stochastic frontier function [21,22]. This study applies a self-dual Cobb-Douglas SFA Method. It needs to assume a functional form for the production technology and the distribution of the technical inefficiency term. The economic, technical, and allocative efficiencies are computed from it's dual cost frontier model. The Cobb-Douglas stochastic frontier specified for this study is as follows:

$$\ln \tilde{y}_i = \beta_0 + \sum_{k=1}^{8} \ln x_{ik} \ln x_{ik} + v_i - u_i \text{ , } i = 1,\ 2, \ldots, 300 \text{ number of farms} \qquad (1)$$

where $\ln$: the natural logarithm, x_1: land, x_2: labor, x_3: tilling, x_4: seed, x_5: fertilizer, x_6: irrigation, x_7: pesticide, x_8: vitamin, β_0: technical efficiency level, $\beta_1, ..., \beta_8$: coefficients of inputs concerning output level, v_i: the random error that farmers cannot control (weather, pests, diseases, measurement error in the output variable, etc.), u_i: the non-negative random error

measures the technical inefficiency relative to the stochastic frontier, $\tilde{y}_i$: the farms observed output adjusted for the stochastic random noise captured by v_i. For $u_i = 0$ the farm lies on the Stochastic Production Function and $u_i > 0$ represents inefficiency in the farm. It is assumed that $Cov(v_i, u_i) = 0$, $Cov(v_i, x_i) = 0$, and $Cov(u_i, x_i) = 0$. The variance parameters of the model are expressed as: $\sigma^2 = \sigma_v^2 + \sigma_u^2$, $\gamma = \frac{\sigma_u^2}{\sigma^2}$, $0 \le \gamma \le 1$, where, $\gamma = 0$: absence of stochastic technical inefficiency when stochastic frontier model becomes a average frontier model, and $\gamma = 1$: the stochastic random error term is absent when the stochastic frontier model becomes a full frontier model [23]. The parameter $\lambda = \frac{\sigma_u}{\sigma_v}$ is a measure of the comparative variability of two inefficiency sources and $\lambda^2 \to 0$ implies that $\sigma_v^2 \to \infty$ and/or $\sigma_u^2 \to 0$ means that the random shocks dominate in explaining the inefficiency. When $\sigma_v^2 \to 0$ then, the gaps to the frontier are due to technical inefficiency.

The ith farm-specific technical efficiency is the ratio of y_i and y^*, given the input levels and can be written as $TE_i = \frac{y_i}{y_i^*} = \frac{f(x_i, \beta)e^{v_i - u_i}}{f(x_i, \beta)e^{v_i}} = e^{-u_i}$, $0 \le TE_i \le 1$, where y_i is the observed output of ith farm, y^* is the corresponding frontier output, u_i are non-negative truncations of the $N(\mu, \sigma_u^2)$ distribution and $\mu = z_i \delta_i$ where z_i is a $(k \times 1)$ vector of variables that influence efficiency and δ_i to be estimated $(1 \times k)$ vector of parameters.

Equation (1) constitutes the basis for obtaining the technically efficient input vector x_{ik}^T. The dual stochastic frontier cost function model is analytically derived from the stochastic production model. The economically efficient input vector x_{ik}^T is derived from the dual stochastic frontier cost function. The dual stochastic frontier cost function model is

$$C(p_{ik}, \tilde{y}_i) = \alpha_0 \prod_{k=1}^{8} p_{ik}^{\beta_{ik}\alpha_{ik}} \tilde{y}_i^{\alpha_{ik}} \qquad (2)$$

where, $C\left(p_{ik},\tilde{y}_i\right)$ is the cost function, $\alpha_0 = \left(\frac{1}{\beta_0^{\alpha_{ik}}}\right)\left(\frac{\sum_{k=1}^{8}\beta_{ik}}{\prod_{k=1}^{8}\beta_{ik}^{\beta_{ik}\alpha_{ik}}}\right)$ and $\alpha_{ik} = \frac{1}{\sum_{k=1}^{8}\beta_{ik}}$.

Differentiating (2) with respect to each input's price and applying Shephard lemma the system of input demand function can be written as:

$$x_{ik}^E = \frac{\partial C\left(p_{ik},\tilde{y}_i\right)}{\partial p_{ik}} = \alpha_0\left(\beta_{ik}\alpha_{ik}\right)\prod_{k=1}^{8}\frac{1}{p_{ik}}\,p_{ik}^{\beta_{ik}\alpha_{ik}}\,\tilde{y}_i^{\alpha_{ik}}. \qquad (3)$$

From the result of stochastic frontier production function (1), we can get the technically efficient input vector x_{ik}^T. Multiplying the observed input vector x_{ik}, the technically efficient input vector x_{ik}^T and the economically efficient input vector x_{ik}^E by the input price vector provides the observed, technically efficient, and economically efficient costs of production of the i-th farm equal to $p_{ik}x_{ik}$, $p_{ik}x_{ik}^T$, and $p_{ik}x_{ik}^E$ respectively which compute the TE, AE, and EE indices for the ith farm as:

$$TE = \frac{p_{ik}x_{ik}^T}{p_{ik}x_{ik}},\ AE = \frac{p_{ik}x_{ik}^E}{p_{ik}x_{ik}^T},\ \text{and}\ EE = \frac{p_{ik}x_{ik}^E}{p_{ik}x_{ik}}.$$

Data Envelopment Analysis (DEA) Model

The DEA is a linear programming method used to formulate a piece-wise linear surface over the input and output data points. The linear programming problems are solved for each farm in the sample to construct the frontier surface and produce the level of inefficiency. The level of inefficiency is the gap between the frontier and the observed data point. The efficient farm with an efficiency score of one lies on the production frontier, and the inefficient farm with an efficiency score of less than one exists beneath the frontier. Charnes et al., (1978) proposed an input orientation DEA method that assumed constant returns to scale (CRS) [24]. Moreover, Banker et al., (1984) assumed variable returns to scale (VRS) in the DEA model [25]. Since this study considers potatoes, a single output and eight inputs it selects the input orientation model. This model asses how much input can be changed proportionally to produce a fixed amount of output.

Determinants of Inefficiency

The following equation presents the inefficiency effects model. For the SFA method, technical efficiency is calculated in a single-stage method in which the technical inefficiency effects are modeled as a function of socio-economic characteristics and infrastructure factors. The DEA method estimates inefficient effects using the Tobit regression model.

$$IE_i = \delta_0 + \delta_1 z_{i1} + \delta_2 z_{i2} + \delta_3 z_{i3} + \delta_4 z_{i4} + \delta_5 z_{i5} + \delta_6 z_{i6} + \delta_7 z_{i7} + \delta_8 z_{i8} + \delta_9 z_{i9} + w_i \quad (4)$$

where, IE_i : inefficiency, z_1 : age (year), z_2 : education (years of schooling), z_3 : experience (year), z_4 : land fragmentation (number of plots), z_5 : family size, z_6 : deweeding, z_7 : access to credit (dummy), z_8 : cold storage (dummy), z_9 : training (dummy), and w_i : error term.

Results and Discussion

Stochastic Frontier Analysis

Initially, the authors estimate the SFA model using the maximum likelihood method and Table 1 shows results of the Cobb-Douglas stochastic frontier model with technical inefficiency factors.

Table 1: Results of Cobb-Douglas stochastic frontier model

**Variables**	**Parameters**	**Coefficients**
Constant	β_0	2.799
ln of land	β_1	0.575**
ln of labor cost	β_2	0.337**
ln of tilling cost	β_3	0.404*
ln of seed cost	β_4	0.491*
ln of fertilizer cost	β_5	0.285**
ln of irrigation cost	β_6	0.152*
ln of pesticide cost	β_7	0.126*
ln of vitamin cost	β_8	0.068*
**Inefficiency Variables**		
Constant	δ_0	4.792
Age	δ_1	-0.524*
Education	δ_2	-0.377**
Experience	δ_3	-0.256**

Land fragmentation	δ_4	0.338*
Family size	δ_5	0.097*
Deweeding	δ_6	-0.297*
Access to credit (dummy)	δ_7	-0.383*
Cold storage (dummy)	δ_8	-0.212*
Training (dummy)	δ_9	-0.432*
**Variance Parameters**		
$\sigma^2 = \sigma_v^2 + \sigma_u^2$		0.064
$\gamma = \frac{\sigma_u^2}{\sigma^2}$		0.853
σ_v^2		0.009
σ_u^2		0.055
Log likelihood		324.670

Note: *: p<0.05, **: 0.05<p<0.01.

The results show that the coefficients of eight variables are positive and statistically significant at 5% significance level. It indicates that eight variables are important to determine potato production. The elasticity is highest for land. Farmers are not always using their total land for potato cultivation. After the acquisition of land, the land should be prepared well by tilling may be by the cow or by the tractors. The farmers have to have good quality seeds no matter whether the seeds are local or HYV varieties. Without good quality seeds, production would be hampered since good quality seeds are prerequisites for good production. The farmers have to use good quality fertilizer no matter if they grow local or HYV varieties. Moreover, without good quality fertilizer, production would be hampered which is a prerequisite for boosting production. It is observed that irrigation; pesticides and vitamins have a relatively small effect [Table 1].

The estimated δ-coefficients of the explanatory variables indicate that the inefficiency variables of farms significantly contribute to explain the technical inefficiency effects in potato farming. The signs of estimated coefficients tell us that these variables cause variation in the technical efficiency of potato farms and affect the capability of farms in adequately utilizing existing technology and Infrastructure. The return to scale $\left(\sum_{i=1}^{8} \beta_i = 2.44 \right)$ indicates increasing returns to

scale in potato farming. The estimated value of γ parameter is 0.853 and the variance parameter σ^2 is 0.06 in the stochastic frontier. All coefficients of inefficiency factors are statistically significant at 5% level indicating that there are inefficiency effects in the potato farming in the sample farms and the random factors of the inefficiency effects significantly contribute $\left(\sigma_u^2 = 0.055\right)$ in potato farming efficiency. That is the technical inefficiency effects are important components to determine the variability and level of potato production. The negative coefficients of education, age, and experience imply farmers with more years of schooling, experience, and age are more technically efficient. Since weed hampers potato production farmers need to de-weed their land for getting more output. As far as land fragmentation is concerned, farmers with smaller plot sizes are more technically inefficient. The higher the smaller plots to be managed it would be difficult to manage the land and especially irrigation and tillage would be difficult with a tractor and water pump with electricity. Since farmers have smaller pieces of land they don't use very much technologically advanced methods of cultivation. The positive coefficient of the family size implies that the higher the family size, the higher the inefficiency though the coefficient is not significant at 5% level.

Input- Oriented DEA Frontier

Input-oriented DEA frontiers with CRS and VRS are computed. The ratio of CRS and VRS efficiency estimates the scale efficiency of potato farming. The frequency distribution of farms according to technical and scale efficiencies are presented in Supplementary Table 1. It is observed that most of the farms (87%) have technical efficiency scores within (1-70) % efficiency index. Average scores of technical efficiency for CRS and VRS TEs are almost closer. About 83% of farms fall in (70-100) % scale efficiency index. In terms of scale economies, 54% of farms have increasing, 22% have constant and (24%) have decreasing returns to scale [Supplementary Table 1]. According to Silberberg and Suen, (2000) farms using the same technology will have increasing return to scale with relatively low output, decreasing return to scale with relatively high output and constant returns to scale when the output level is equal to mean output [26].

Estimated Production, Cost and Input Demand Functions

The dual cost frontier is estimated from the stochastic production frontier for the inefficiency components. The stochastic production function and frontier cost function are obtained using the findings presented in Table 1.

Stochastic Production Function is as follows:

$$y_i = 2.799\, x_{i1}^{0.575} x_{i2}^{0.337} x_{i3}^{0.404} x_{i4}^{0.491} x_{i5}^{0.285} x_{i6}^{0.152} x_{i7}^{0.126} x_{i8}^{0.068}, \text{ where } i = 1, 2, ..., 300.$$

Dual cost frontier function is logically estimated as follows:

$$C\left(p_{ik}, \tilde{y}_i\right) = 4.435\, P_{i1}^{0.236} P_{i2}^{0.138} P_{i3}^{0.166} P_{i4}^{0.201} P_{i5}^{0.117} P_{i6}^{0.062} P_{i7}^{0.052} P_{i8}^{0.028} \tilde{y}_i^{0.410}, \text{ where } i = 1, 2, ..., 300.$$

Input demand function can be written as

$$x_{i1} = \frac{1.05 p_{i2}^{0.138} p_{i3}^{0.166} p_{i4}^{0.201} p_{i5}^{0.117} p_{i6}^{0.062} p_{i7}^{0.052} p_{i8}^{0.028} \tilde{y}^{0.41}}{p_{i1}^{0.764}}, \text{ where } i = 1, 2, ..., 300.$$

Economic, Technical and Allocative Efficiencies by SFA and DEA method

The frequency distribution (%) of economic, technical, and allocative efficiency scores of farms for both methods are shown in Supplementary Table 2. In the SFA method, most of the farms 89% are technologically efficient and falls (70-100%) efficiency class while 11% of farms fall (1-69%) efficiency class. In the case of AE, 68% of farms fall (70-100%) efficiency class, and 32% of farms fall in (1-69%) efficiency class. In the case of EE, 36% of farms fall (70-100%) efficiency class, and 64% of farms fall in (1-69%) efficiency class. Farms have considerable variations in efficiency. The calculated mean of economic, technical, and allocative efficiencies indicates that there are considerable economic, technical, and allocative inefficiencies in potato farming and production can be increased through efficiency improvement. It is seen that TE has the highest mean value and EE has the lowest mean value. Moreover, Supplementary Table 2 shows the frequency distribution (%) and summary statistics of economic, technical, and allocative efficiencies of CRS and VRS DEA frontier methods. The results of CRS DEA and VRS DEA frontiers reveal that farmers can improve production efficiency without applying more advanced or new technology in the production process which reduces production costs. Allocative efficiency is greater than the technical and economic efficiencies in potato farming.

Comparison between the Results of SFA and DEA Methods

This study has an objective to compare efficiency measures from SFA and DEA methods and to assess whether any significant differences in the estimates of the efficiency. These two methods are different in explaining the gap between the estimated function and observations. To explain the gap SFA method considers both producers' inefficiency and some random elements which are not under the owner's control, but the DEA method supposes the absence of random error and considers only the producer's inefficiency. A previous study pointed out that the empirical result of both SFA and DEA methods, two quite different approaches of efficiency estimation may differ for differences in the choice of input and output variables, characteristics of data analyzed, estimation procedures, and measurement & specification errors [27]. If results are similar, then the measure of efficiency and relative efficiency in terms of socio-economic characteristics and infrastructure factors are robust and can be used as a basis for policy recommendation. For the SFA the efficiency score is 85.3, however, in the case of DEA, technical efficiency for CRS and VRS are 43.76 and 52.71 respectively and scale efficiency is 84.5. In stochastic frontier analysis, it is assumed that all farms have the estimated efficiency scores. But in data envelopment analysis each farm has different efficiency scores. The DEA method represents the average efficiency scores of all farms. From the efficiency score, it is seen that VRS technical efficiency has a higher value which implies that VRS TE enveloped data more closely than CRS TE. The efficiency score of the SFA method is higher than the DEA method.

Returns to Scale

The stochastic frontier analysis estimates returns to scale of farm is $\sum_{i=1}^{8} \beta_i = 2.44$ which is greater than 1. It implies that there is an increasing returns to scale and inefficiency in potato farming. This result is assumed for all farms. Data envelopment analysis estimates returns to scale for all farms separately: 54% of farms have increasing, 22% of farms have constant, and 24% of farms have decreasing return to scale. It is easy to understand which farm has what types of returns to scale. The majority of the farms have increasing returns to scale which has a similar interpretation as SFA that there is inefficiency in potato farming. The following Figure 1 illustrates the mean of economic (EE), technical (TE), and allocative efficiencies (AE) estimates of SFA and DEA methods.

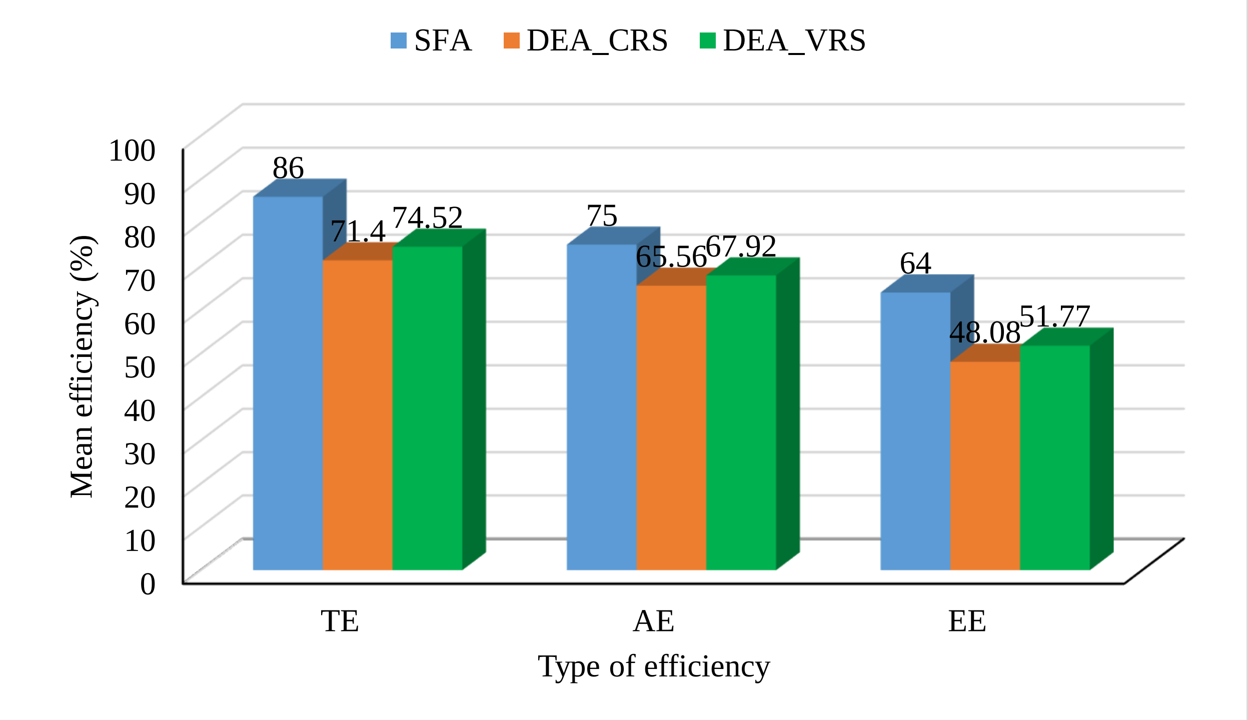

Figure 1: Mean efficiency of economic, technical and allocative obtained by SFA and DEA methods

The mean values of efficiency estimates based on Stochastic Frontier Analysis are higher than those based on the CRS and VRS DEA frontier [Figure 1]. It implies that the stochastic frontier is well-fitted to the data set compared to the DEA frontier. Technical efficiency scores of the SFA model are larger than both CRS and VRS DEA models. A previous study also got similar findings for the swine industry in Hawaii [28]. The value of standard deviations shows that the DEA frontier shows larger variability in economic, technical, and allocative efficiency estimates than the stochastic frontier.

Factors affecting inefficiency from SFA and DEA method

In order to make an appropriate decision, it is crucial to understand the various aspects that affect a company's efficiency. The factors taken into consideration in this research that impact the economic, technical, and allocative efficiencies of SFA and DEA approaches are shown in Table 2.

Table 2: Coefficients of factors affecting inefficiency

Factors	Stochastic Frontier Analysis (SFA)			Data Envelopment Analysis					
				CRS (Overall TE)			VRS (Pure TE)		
	TI	AI	EI	TI	AI	EI	TI	AI	EI
Constant	1.79	0.75	0.63	0.58	0.73	0.48	0.82	0.64	0.55
Age	-0.52*	-0.05*	-0.01*	-0.03*	-0.01*	-0.02*	-0.05*	-0.01*	-0.01*
Education	-0.38*	-0.03*	-0.02*	-0.02*	-0.06*	-0.04*	-0.03*	-0.09*	-0.03*
Experience	-0.26*	-0.04*	-0.01*	-0.06*	-0.03*	-0.07*	-0.08*	-0.04*	-0.10*
Land fragmentation	0.34*	0.02*	0.03*	0.02*	0.01*	0.01*	0.06*	0.06*	0.02*
Family size	0.097*	0.009*	0.006*	0.012*	0.002*	0.009*	0.027*	0.002*	0.022*
Deweeding	-0.6*	-0.004*	-0.002*	-0.01*	-0.01*	-0.01*	-0.02*	-0.03*	-0.04*
Access to credit (dummy)	-0.98*	-0.02*	-0.02*	-0.04*	-0.09*	-0.04*	-0.07*	-0.08*	-0.11*
Cold storage (dummy)	-0.01*	-0.015*	-0.017*	-0.13*	-0.05*	-0.12*	-0.06*	-0.03*	-0.13*
Training (dummy)	-1.43*	-0.02*	-0.06*	-0.03*	-0.03*	-0.19*	-0.17*	-0.04*	-0.11*

*Indicate significant at 5% level

The socio-economic and infrastructural factors' results for both methods are similar in direction and different in coefficient values. Table 2 shows that the coefficient of farming experience is negative for all inefficiencies (TI, AI, EI) denoting that households with more farming experience can improve their skill and capability of managing inputs efficiently and tend to technically efficient potato production. The coefficient for the land fragmentation variable is positive, implying that inefficiency tended to increase with the increase in land fragmentation. If lands are more fragmented, those farms are technically less efficient than those of less fragmented farms. The larger land size is more economic because farmers can easily apply modern technologies like tractors and better irrigation management. The coefficient of family size is positive but not significant, implying that large or small family size has not significantly affected farming efficiency. De-weeding also has a significant effect on potato farming efficiency. The coefficient of education is negative and significant suggests that farmers with more schooling tended to be technically more efficient than farmers with lower education or no education at all.

Two methods give different efficiency scores; provide returns to scale in a different ways. However, from both methods, the authors can understand that potato farming is inefficient. From DEA, it can be identified which farm presents which type of returns to scale. In the case of factors affecting inefficiency, the estimated coefficients are different in value but the same in direction. The efficiency score of the SFA model is higher than the DEA model, which implies that the SFA frontier fits tighter than the DEA frontier. The SFA and VRS DEA are almost similar in the case of estimated technical, allocative, and economic efficiency. In the case of DEA, VRS TE enveloped data more closely than CRS TE. The authors may conclude that Stochastic Frontier Analysis is more applicable to the agriculture sector since the owner cannot control random errors that exist in this sector.

Conclusion

Bangladesh achieved commendable success in potato production even under the global context due to favorable agro-climate and lower labor costs. Considering these opportunities, potato farming efficiency can help Bangladesh achieve short- and long-term economic targets. It requires studying the possibility of potato productivity raising through efficiency improvement without introducing new technologies or increasing the resource base. The empirical results of both methods show that the SFA model's efficiency score is higher than the DEA model, which implies that the SFA frontier fits tighter than the DEA frontier. SFA and VRS DEA are almost similar in the case of estimated technical, allocative, and economic efficiency. In the case of DEA, VRS TE enveloped data more closely than CRS TE. The variability and level of production of potato farming are determined by technical inefficiency. The estimated infrastructure and socio-economic factors jointly determine the variabilities of potato production. There are significant economic, technical, and allocative inefficiencies in potato farming and scope to increase potato production levels through efficiency improvement, increasing the farm household's income and welfare. It is concluded that Stochastic Frontier Analysis is more applicable to the agriculture sector because the random error cannot control by the owners in this sector.

This study emphasizes that the following factors need attention to increase potato productivity. Formal education, particularly agriculture-related education, can help farmers increase their knowledge about cultivation and cost-minimizing input use, which can improve allocative

efficiency. An extension program could be used to reorient the application of methods, timing, and amount of inputs and production methods. Land tenure and management policies could be designed to reduce land fragmentation that also can lessen obstacles to utilizing existing technology efficiently and helps better allocation of inputs, utilization of irrigation, fertilizer, and land preparation in a cost-minimizing way since there are no training, cold storage, and access to credit facilities. If the farmers have those facilities, the inefficiency of production performance would be reduced substantially. To achieve production efficiency, more emphasis should be placed on choosing the appropriate technology and efficient use of resources for the scale of operation. Further research would be better if collected data and information were based on a larger sample size.

Acknowledgment

The authors are grateful to the respondents for participating in this study and providing consent to publish the study findings after removing identifiable information.

Conflict of Interest

The authors declare no conflict of interest.

Ethics Statement

Before participating in the survey, the authors explained the purpose of the study to the participants and assured them that their answers would be kept private, that no personally identifiable information would be shared, and that their verbal consent would be obtained.

Data Availability

All relevant data are within the manuscript and some data are available in a Supporting Information file.

Funding

The authors received no specific funding for this work.

References

1. Salan MSA, Hossain MM, Sumon IH, Rahman MM, Kabir MA, Majumder AK. Measuring the impact of climate change on potato production in Bangladesh using Bayesian Hierarchical Spatial-temporal modeling. PLoS One. 2022;17: e0277933. doi:10.1371/journal.pone.0277933

2. Hossain MM, Abdulla F. Forecasting Potato Production in Bangladesh by ARIMA Model. J Adv Stat. 2016;1: 191–198. doi:10.22606/jas.2016.14002
3. Rahim MA, Sultana N, Mannan ATMM, Ahmed N. Chapter 22 - Potato production in Bangladesh. In: Çalişkan ME, Bakhsh A, Jabran KBT-PPW, editors. Potato Production Worldwide. Academic Press; 2023. pp. 397–407. doi:https://doi.org/10.1016/B978-0-12-822925-5.00019-0
4. Nunn N, Qian N. The Potato's Contribution to Population and Urbanization: Evidence from a Historical Experiment. Q J Econ. 2011;126: 593–650.
5. Miah MAM, Hossain TMB, Hossain S, Kadian MS, Hossain M. Farmers' perception about variety development and Abiotic stresses on potato cultivation in Bangladesh. Bangladesh J Agric Res. 2013;38: 401–416. doi:10.3329/bjar.v38i3.16926
6. Singha U, Maezawa S. Production, marketing system, storage and future aspect of potato in Bangladesh. Rev Agric Sci. 2019;7: 29–40. doi:10.7831/ras.7.29
7. Hossain MM. Technical Efficiency Measurement of Green Chili Production in Bogra District of Bangladesh. J Stat Appl Probab Lett. 2016;3: 97–101. doi:10.18576/jsapl/030204
8. Hossain MM, Alam MA, Kamal Uddin M. Application of Stochastic Frontier Production Function on Small Banana Growers of Kushtia District in Bangladesh. J Stat Appl Probab An Int J. 2015;4: 337–342. doi:10.12785/jsap/040218
9. Wadud MA. Farm efficiency in Bangladesh. Newcastle University. 1999. Available: http://theses.ncl.ac.uk/jspui/handle/10443/184
10. Wadud A, White B. Farm household efficiency in Bangladesh: a comparison of stochastic frontier and DEA methods. Appl Econ. 2000;32: 1665–1673. doi:10.1080/000368400421011
11. Wadud MA. Technical, Allocative, and Economic Efficiency of Farms in Bangladesh: A Stochastic Frontier and DEA Approach. J Dev Areas. 2003;37: 109–126.
12. Chowdhury MKI, Rumi SF, Rahman MM. Efficiency of Rice Farms during Boro Period in Bangladesh:An Econometric Approach | Enhanced Reader. Glob J Manag Bus Res Financ . 2013;13: 33–44.
13. Hossain MK, Kamil AA, Baten MA, Mustafa A. Stochastic Frontier Approach and Data Envelopment Analysis to Total Factor Productivity and Efficiency Measurement of Bangladeshi Rice. PLoS One. 2012;7: e46081. Available:

https://doi.org/10.1371/journal.pone.0046081
14. Johansson H. Technical, Allocative, and Economic Efficiency in Swedish Dairy Farms: The Data Envelopment Analysis Versus the Stochastic Frontier Approach. 2005 International Congress, August 23-27, 2005, Copenhagen, Denmark. 2005. pp. 1–18. doi:10.22004/AG.ECON.24478
15. Islam KMZ, Hoque MN. Application of Stochastic Frontier and Data Envelopment Analysis on the Measurement of Farming Efficiencies in Bangladesh. North South Bus Rev. 2014;5: 47–68.
16. Umar HS, Girei AA, Kachiro SL. Comparison of data envelopment and stochastic frontier models in analysis of efficiency and its factors in ginger farms in Kaduna State, Nigeria. J Agric Res Dev. 2018;16: 11–21. doi:10.4314/jard.v16i2.2
17. Abdulai S, Nkegbe P, Donkoh SA. Do data envelopment and stochastic frontier analyses produce similar efficiency estimates? The case of Ghanaian maize production. African J Agric Resour Econ. 2018;13: 251–263. Available: https://econpapers.repec.org/RePEc:ags:afjare:284994
18. Rabbany MG, Mehmood Y, Hoque F, Sarker T, Hossain KZ, Khan AA, et al. Do credit constraints affect the technical efficiency of Boro rice growers? Evidence from the District Pabna in Bangladesh. Environ Sci Pollut Res Int. 2022;29: 444–456. doi:10.1007/S11356-021-15458-1
19. Salam MA. Exploring the seasonal yield variability, production risk and efficiency: the case of rice farms in Bangladesh. Heliyon. 2022;8: e10559. doi:10.1016/j.heliyon.2022.e10559
20. Farrell MJ. The Measurement of Productive Efficiency. J R Stat Soc Ser A. 1957;120: 253–290. doi:10.2307/2343100
21. Aigner D, Lovell CAK, Schmidt P. Formulation and estimation of stochastic frontier production function models. J Econom. 1977;6: 21–37. doi:10.1016/0304-4076(77)90052-5
22. Meeusen W, van den Broeck J. Efficiency Estimation from Cobb-Douglas Production Functions with Composed Error. Int Econ Rev (Philadelphia). 1977;18: 435–444.
23. Aigner DJ, Chu SF. On Estimating the Industry Production Function. Am Econ Rev. 1968;58: 826–839.
24. Charnes A, Cooper WW, Rhodes E. Measuring the efficiency of decision making units. Eur

J Oper Res. 1978;2: 429–444. doi:https://doi.org/10.1016/0377-2217(78)90138-8

25. Banker RD, Charnes A, Cooper WW. Some Models for Estimating Technical and Scale Inefficiencies in Data Envelopment Analysis. Manage Sci. 1984;30: 1078–1092. doi:10.1287/mnsc.30.9.1078
26. Silberberg E, Suen WC. The Structure of Economics: A Mathematical Analysis. 3rd editio. McGraw-Hill; 2000.
27. Sharma KR, Leung P, Zaleski HM. Technical, allocative and economic efficiencies in swine production in Hawaii: a comparison of parametric and nonparametric approaches. Agric Econ. 1999;20: 23–35. doi:https://doi.org/10.1016/S0169-5150(98)00072-3
28. Sharma KR, Leung P, Zaleski HM. Productive Efficiency of the Swine Industry in Hawaii: Stochastic Frontier vs. Data Envelopment Analysis. J Product Anal. 1997;8: 447–459. doi:10.1023/A:1007744327504

Click here to access/download
Supporting Information
Supplementary Tables.docx

Sample data

Click here to access/download
Supporting Information
Sample Data_12.02.2023.xlsx
