# Supplementary material for: Estimating the potato farming efficiency: A comparative study between stochastic frontier analysis and data envelopment analysis
Source: PLoS One. 2023 Apr 13;18(4):e0284391. doi: 10.1371/journal.pone.0284391 (PMC10101415; doi:10.1371/journal.pone.0284391)
Supplement: Attachment — Submitted filename: Response to Reviewers.pdf [file pone.0284391.s005.pdf]

Author responses to the review comments:

We would like to express our sincere gratitude to the reviewers and the Editors for their valuable comments. We have considered all the comments made by the reviewers and thoroughly revised and formatted the manuscript accordingly. A detailed response to each of the comments is provided in the table below:

**Academic Editor comments:**	**Response**	**Note**
Please include the following items when submitting your revised manuscript:<br>A rebuttal letter that responds to each point raised by the academic editor and reviewer(s). You should upload this letter as a separate file labeled 'Response to Reviewers'.<br>A marked-up copy of your manuscript that highlights changes made to the original version. You should upload this as a separate file labeled 'Revised Manuscript with Track Changes'.<br>An unmarked version of your revised paper without tracked changes. You should upload this as a separate file labeled 'Manuscript'.<br>If you would like to make changes to your financial disclosure, please include your updated statement in your cover letter. Guidelines for resubmitting your figure files are available below the reviewer comments at the end of this letter.	Thank you very much for carefully checking the manuscript and providing insightful comments.<br>All required files are uploaded to the journal system.	Revised texts are in red color.
**Journal Requirements:**	**Response**	**Note**
When submitting your revision, we need you to address these additional requirements.<br>1. Please ensure that your manuscript meets PLOS ONE's style requirements, including those for file naming. The PLOS ONE style templates can be found at<br>https://journals.plos.org/plosone/s/file?id=wjVg/PLOSOne_formatting_sample_main_body.pdf and<br>https://journals.plos.org/plosone/s/file?id=ba62/PLOSOne_formatting_sample_title_authors_affiliations.pdf	Thanks. We revised the manuscript following the PLOS ONE style.	Revised texts are in red color.
2. You indicated that ethical approval was not necessary for your study. We understand that the framework for ethical oversight requirements for studies of this type may differ depending on the setting and we would appreciate some further clarification regarding your research. Could you please provide further details on why your study is exempt from the need for approval and confirmation from your institutional review board or research ethics committee (e.g., in the	Thanks. We add the consent form as an "Other" file.	Revised texts are in red color.<br>Page: 4

form of a letter or email correspondence) that ethics review was not necessary for this study? Please include a copy of the correspondence as an ""Other"" file.		
3. In your Data Availability statement, you have not specified where the minimal data set underlying the results described in your manuscript can be found. PLOS defines a study's minimal data set as the underlying data used to reach the conclusions drawn in the manuscript and any additional data required to replicate the reported study findings in their entirety. All PLOS journals require that the minimal data set be made fully available. For more information about our data policy, please see http://journals.plos.org/plosone/s/data-availability.<br>Upon re-submitting your revised manuscript, please upload your study's minimal underlying data set as either Supporting Information files or to a stable, public repository and include the relevant URLs, DOIs, or accession numbers within your revised cover letter. For a list of acceptable repositories, please see http://journals.plos.org/plosone/s/data-availability#loc-recommended-repositories. Any potentially identifying patient information must be fully anonymized.<br>Important: If there are ethical or legal restrictions to sharing your data publicly, please explain these restrictions in detail. Please see our guidelines for more information on what we consider unacceptable restrictions to publicly sharing data: http://journals.plos.org/plosone/s/data-availability#loc-unacceptable-data-access-restrictions. Note that it is not acceptable for the authors to be the sole named individuals responsible for ensuring data access.<br>We will update your Data Availability statement to reflect the information you provide in your cover letter.	Thanks. We add the following. All relevant data are within the manuscript and data are available in a Supporting Information file (S3 Data).	Revised texts are in red color.<br>Page: 18
4. Your ethics statement should only appear in the Methods section of your manuscript. If your ethics statement is written in any section besides the Methods, please delete it from any other section.	Thanks. We moved it to the Methods section.	Revised texts are in red color.<br>Page: 4
5. Please include captions for your Supporting Information files at the end of your manuscript, and update any in-text citations to match accordingly. Please see our Supporting Information guidelines for more information:	Thanks. We add captions of the Supporting Information files at the end of your manuscript.	Revised texts are in red color.<br>Page:17-18

http://journals.plos.org/plosone/s/supporting-information.		
6. Please review your reference list to ensure that it is complete and correct. If you have cited papers that have been retracted, please include the rationale for doing so in the manuscript text, or remove these references and replace them with relevant current references. Any changes to the reference list should be mentioned in the rebuttal letter that accompanies your revised manuscript. If you need to cite a retracted article, indicate the article's retracted status in the References list and also include a citation and full reference for the retraction notice	Thanks. We checked the reference list and ensure that it is complete and correct. We use Mendeley for citation.	
**Reviewer 1 comments:**	**Response**	**Note**
My comments are in the file.<br>1-The potato (Solanum tuberosum L.) is the most productive crop and the third-largest source of food on earth [1]. (The following information is also available in the literature. Potato is in the fifth place after sugarcane, corn, rice and wheat in the list of the most produced plant products in the world. Please refer to the literature: FAOSTAT (2022). World Production Quantities of Crops. http://www.fao.org/faostat/en/#data/QC. and Kadakoğlu, B., Karlı, B. (2021). Economic Analysis of Potato Production in Afyonkarahisar Province, KSU J. Agric Nat 25 (3): 581-588. https://doi.org/10.18016/ksutarimdoga.vi.947387)	Thank you very much for carefully checking the manuscript and providing insightful comments.<br><br>We revise it and add the suggested citations.	Revised texts are in red color.<br>Page: 2 (Ref. 2, 3)
2-To the best of our knowledge, no research has been done yet on potato farming efficiency using both SFA and DEA. (On the contrary, a study was conducted in Türkiye on the technical efficiency of potato cultivation using both SFA and DEA. According to the results of the research, SFA 69.0, DEA-CRS 76.4, DEA-VRS 84.6 were found. Please refer to the literature: Kadakoğlu, B. (2021). Analysis of Technical and Economic Efficiency of Potato Production in Afyonkarahisar Province, Isparta University of Applied Sciences, The Institute of Graduate Education Department of Agricultural Economics, M.Sc. Thesis. http://dx.doi.org/10.13140/RG.2.2.20260.96641)	Thanks for highlighting this point.<br>We have revised the manuscript as per your guidelines.	Revised texts are in red color.<br>Page: 3 (Ref. 22)
3-This study applies a self-dual Cobb-Douglas SFA Method. (What is your reason for choosing this method? As you know, there are statistics for Cobb-Douglas or Translog method in SFA and as a result, the method is selected. Please apply the statistics and choose your method accordingly.)	We appreciate your comments. We revise the manuscript.<br>The Cobb-Douglas type production function has been used extensively by researchers worldwide and the existing literature motivates us to use the	Revised texts are in red color.<br>Page: 5

	self-dual Cobb-Douglas SFA Method in this study.	
4-Results of Cobb-Douglas stochastic frontier model. (It is recommended to compare your study result with another study on the same method and the same product. Please refer to the literature: Kadakoğlu, B., Karlı, B. (2022). Technical Efficiency of Potato Production in Turkey by Stochastic Frontier Analysis,Custos e Agronegocio, 18(2), 163-178. https://www.researchgate.net/publication/364303318_Technical_Efficiency_of_Potato_Production_in_Turkey_by_Stochastic_Frontier_Analysis)	Thanks. We have revised the Result section according to your guidelines and feedback.	Revised texts are in red color.<br>Page: 7-11
5-Further research would be better if collected data and information were based on a larger sample size. (Why didn't you increase the sample size of the research more and make it more comprehensive? Include the constraints of your research.)	Thank you very much. We add a limitation section as per your comments.	Revised texts are in red color.<br>Page: 16
6-References (It is recommended to include the above-mentioned references to increase the scope of the research.)	We are thankful to you for carefully checking the manuscript. We revised the citation and reference list.	Revised texts are in red color.<br>Page: 18-22
**Reviewer 2 comments:**	**Response**	**Note**
Summary<br>This study first assesses whether SFA is a better method than DEA in estimating potato farming efficiency. Second, this study identifies the determinants of potato farming efficiency.<br>I only have a few minor comments.	Thanks. We appreciate your comments. We have revised this section as per your feedback.	Revised texts are in red color.
Abstract<br>Please rewrite the abstract. The current abstract is quite lengthy and repetitive.<br>Data<br>Please provide descriptive statistics of variables used in this study.<br>Please provide references for the inputs and outputs used.<br>Results<br>For the analysis of efficiency determinants, please provide references to each finding.<br>References<br>Please keep the references consistent.<br>Other comments<br>English writing is relatively poor, for example, "where Tk. Is the currency of Bangladesh". Please approach professional editing services before resubmitting your manuscript. Again, the author(s) could benefit from a professional proofreader.	Thank you very much. This section is revised as per the comment.<br>We revise the abstract of this manuscript.<br><br>We add the descriptive statistics of variables used in this study.<br><br>We add the references for efficiency determinants.<br><br>We use Mendeley for citation and follow the PLOS ONE style. We check and correct all typos and grammatical mistakes.	Revised texts are in red color.<br>Page: 1-2, 4, 7-8, 10-11, 12-13

Finally, the revised manuscript has been produced following the valuable comments and suggestions of the reviewers. Once again, we would like to thank the reviewers for their sincere dedication, professional insights, and earnest cooperation in reviewing the manuscript.
