# Supplementary material for: Estimating the potato farming efficiency: A comparative study between stochastic frontier analysis and data envelopment analysis
Source: PLoS One. 2023 Apr 13;18(4):e0284391. doi: 10.1371/journal.pone.0284391 (PMC10101415; doi:10.1371/journal.pone.0284391)
Supplement: Attachment — Submitted filename: Response to Reviewers.docx [file pone.0284391.s006.docx]

**Author responses to the review comments:**

We would like to express our sincere gratitude to the reviewers and the Editors for their valuable comments. We have considered all the comments made by the reviewers and thoroughly revised and formatted the manuscript accordingly. A detailed response to each of the comments is provided in the table below:

| **Academic Editor comments:** | **Response** | **Note** |
| --- | --- | --- |
| Besides the comments from Reviewer 2, I suggest the authors to review some missing literature on agriculture efficiency, although not on potato farms, around the references 9 and 10 on page 3 as follows.  Fuwa, N., Edmonds, C., & Banik, P. (2007). Are small-scale rice farmers in eastern India really inefficient? Examining the effects of microtopography on technical efficiency estimates. Agricultural Economics, 36(3), 335-346. https://doi.org/10.1111/j.1574-0862.2007.00211.x  Asadullah, M. N., & Rahman, S. (2009). Farm productivity and efficiency in rural Bangladesh: the role of education revisited. Applied Economics, 41(1), 17-33. https://doi.org/10.1080/00036840601019125  Madau, F. A. (2011). Parametric Estimation of Technical and Scale Efficiencies in the Italian Citrus Farming. Agricultural Economics Review, 12, 91-111.  Madau, F. A., Furesi, R., & Pulina, P. (2017). Technical efficiency and total factor productivity changes in European dairy farm sectors. Agricultural and Food Economics, 5(1), 17. https://doi.org/10.1186/s40100-017-0085-x  Nguyen, H.-D., Ngo, T., Le, T., Ho, H., & Nguyen, H. T. (2019). The Role of Knowledge in Sustainable Agriculture: Evidence from Rice Farms’ Technical Efficiency in Hanoi, Vietnam. Sustainability, 11(9), 2472.  Rada, N. E., & Fuglie, K. O. (2019). New perspectives on farm size and productivity. Food Policy, 84, 147-152. https://doi.org/10.1016/j.foodpol.2018.03.015 | Thank you very much for completing the second round review process and providing feedback. We believe that the suggestion is helpful to improve the quality of the manuscript.  We appreciate your suggestion. We revised the manuscript in light of the suggested papers and cite them. | Revised texts are in red color.  Page: 3, Ref. [11-16]. |
| Please include the following items when submitting your revised manuscript:  A rebuttal letter that responds to each point raised by the academic editor and reviewer(s). You should upload this letter as a separate file labeled 'Response to Reviewers.  A marked-up copy of your manuscript that highlights changes made to the original version. You should upload this as a separate file labeled 'Revised Manuscript with Track Changes'.  An unmarked version of your revised paper without tracked changes. You should upload this as a separate file labeled 'Manuscript'.  If you would like to make changes to your financial disclosure, please include your updated statement in your cover letter. Guidelines for resubmitting your figure files are available below the reviewer comments at the end of this letter. | Thank you very much for carefully checking the manuscript and providing insightful comments.  All required files are uploaded to the journal system. | Revised texts are in red color. |
| **Journal Requirements:** | **Response** | **Note** |
| 1. Please review your reference list to ensure that it is complete and correct. If you have cited papers that have been retracted, please include the rationale for doing so in the manuscript text, or remove these references and replace them with relevant current references. Any changes to the reference list should be mentioned in the rebuttal letter that accompanies your revised manuscript. If you need to cite a retracted article, indicate the article’s retracted status in the References list and also include a citation and full reference for the retraction notice | Thanks. We checked the reference list and ensure that it is complete and correct. We use Mendeley for citation. |  |
| **Reviewer 2 comments:** | **Response** | **Note** |
| Thank you for giving me a chance to read your work again.  Although the authors have addressed all my comments, I still see several grammar errors there.  For example, pp.10 " The cost on seed is positively and significantly influenced the potato production and this findings is supported by a previous study [16]." or "Moreover, without good quality fertilizer, production would be hampered which is a prerequisite for boosting production which is consistent with other study findings [16]."  In the abstract, "Therefore, the authors aimed to evaluate farm-level efficiency and inefficiency of potato farming in Bangladesh." I think that a word "inefficiency" is unnecessary. | Thanks. We appreciate your comments. We have revised the manuscript as per your feedback. | Revised texts are in red color.  Page: 1, 10 |

Finally, the revised manuscript has been produced following the valuable comments and suggestions of the reviewers. Once again, we would like to thank the reviewers for their sincere dedication, professional insights, and earnest cooperation in reviewing the manuscript.
